# Adrenal response to competitive singing: glucocorticoid metabolites in male *Saltator similis* (Aves, Thraupidae)

**DOI:** 10.1093/conphys/coaf004

**Published:** 2025-02-13

**Authors:** Carolina Lorieri-Vanin, Heriberto Barbosa-Moyano, Claudio de Oliveira Alvarenga, Luís Fábio Silveira

**Affiliations:** Department of Animal Reproduction, School of Veterinary Medicine and Animal Science, University of São Paulo, São Paulo, Brazil; Consultoria Ambiental, Conservare Wild Consulting, São Paulo, Brazil; Department of Animal Reproduction, School of Veterinary Medicine and Animal Science, University of São Paulo, São Paulo, Brazil; Department of Animal Reproduction, School of Veterinary Medicine and Animal Science, University of São Paulo, São Paulo, Brazil; Museum of Zoology of the University of São Paulo, University of São Paulo, São Paulo, Brazil

**Keywords:** Animal welfare, birds, validation, stress

## Abstract

Song competitions involving passerines, such as the Green-winged Saltator (*Saltator similis*), are legally permitted in Brazil and attract widespread participation. This study aimed to assess the adrenal response in male *S. similis* by comparing glucocorticoid metabolite (GCM) levels in uro-faecal extract samples collected during three competitions with those from a rest day (3 days before the competition, D-3), a day before the competition (D-1), the day of the competition (D0) and a day after the competition (D1). Simultaneously, we examined the potential variation in GCM levels among other males not engaged in song competitions but subjected to *ex situ* conditions much like those of participating males. GCM levels were measured using a direct enzyme immunoassay (EIA, CJM006), which was physiologically (ACTH challenge) and analytically validated (parallelism, accuracy and precision tests) for the species under study. The results indicated that the average GCM concentration was lower in the competition group (33.43 ± 22.09 ng/g) as compared to the control group (70.09 ± 29.45 ng/g; *P* = 0.01). However, concentrations spiked significantly on competition day (D0: 38.29 ± 26.12 ng/g) as compared to the rest day (D-3: 28.64 ± 17.86  ng/g; *P* = 0.02), suggesting acute stress response. Given the elevated GCM levels observed during competitions, further research is necessary to confirm the welfare of these birds under competition conditions and to explore the long-term effects of such stressors.

## Graphical Abstract

**Graphical Abstract:**
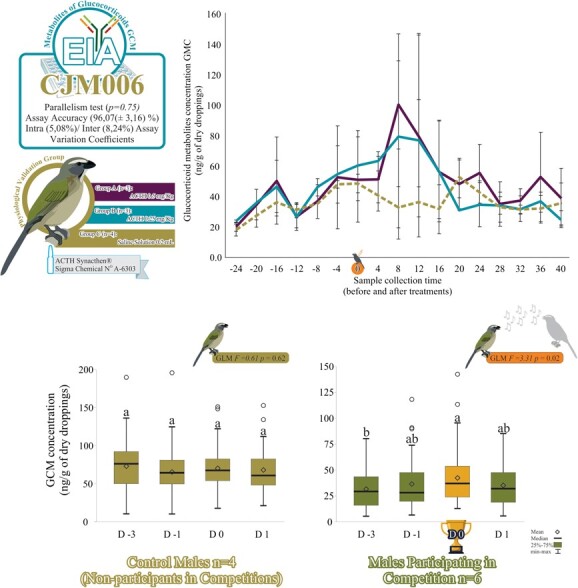

## Highlights

- Peak glucocorticoid metabolite (GCM) excretion occurred 4–8 h post-ACTH injections.
- GCM concentrations reflect endogenous adrenal activity in *Saltator similis*.
- Competition days induced acute stress in *S. similis*, evidenced by increased GCM levels.

## Introduction

Song competitions featuring passerines occur in various parts of the world and are particularly regulated in Brazil under Complementary Law 10/2011 of the Instituto Brasileiro de Meio Ambiente e Recursos Naturais Renováveis (IBAMA). These events are categorized as either quality competitions, where the singing is evaluated, or quantity competitions, where the number of notes achieved in a given time is assessed. Participants, primarily bird breeders, compete for the best vocal performance of their birds. While physical contact between animals is not allowed, acoustic confrontations between males are permitted as a demonstration of territoriality and/or dominance (Kroodsma and Byers, 1991; Slabbekoorn and Smith, 2002; Lyra *et al.*, 2022). However, visual exposure to conspecifics through cages and the inability of the individuals to escape may compromise their welfare.

Although these song competitions are designed to test vocal prowess, they may also present significant challenges to the physiological welfare of the participants. The environments in which animals live are composed of a mix of predictable and unpredictable events (Rich and Romero, 2001; Barbosa-Moyano *et al.*, 2024a). Predictable events include daily variations in light and climatic seasonality, while unpredictable events may involve encounters with other individuals, such as congeners or other species (e.g. predator, prey). Faced with the variability of these events and depending on genetics, experience and life-history stage, animals adjust their vital functions through various physiological processes, referred to as allostasis (McEwen and Wingfield, 2003; Seeley *et al.*, 2022).

The activation of these allostatic systems is intrinsically linked to energy, with the cumulative metabolic demand of the individual’s routine being referred to as allostatic load (McEwen and Wingfield, 2010). When the energy required to cope with events exceeds that available, individuals are said to be in allostatic overload (Landys *et al.*, 2006). The physiological processes of energy acquisition, deposition and mobilization are primarily regulated by glucocorticoids (GCs) (Busch and Hayward, 2009; MacDougall-Shackleton *et al.*, 2019), recognized as among the most significant allostatic mediators (McEwen and Wingfield, 2010). In *ex situ* conditions, wild animals may experience allostatic overload due to constant exposure to unpredictable events and/or aversive stimuli (Morgan and Tromborg, 2007; Seeley *et al.*, 2022). If allostatic overload is persistent, the prolonged actions of mediators such as GCs may result in wear and tear, pathophysiology or damage, rather than protection (McEwen and Wingfield, 2010).

Wild animals under human care must be shielded from the persistence of allostatic overload to mitigate the risk of inducing stereotypies and/or opportunistic diseases, compromising welfare and, in extreme cases, leading to death (Broom, 2011; Nagy-Reis *et al.*, 2019; Barbosa-Moyano *et al.*, 2024a). In this context, the degree of an individual’s well-being is closely tied to their ability to cope with the environment (Broom, 1988). Integrated assessment of well-being is suggested to incorporate three theoretical approaches: the naturalness of living conditions, biological functioning and affective state (Beaulieu, 2024). Therefore, analysis of GCs and/or their metabolites represents one of the widely adopted approaches to assess biological functioning, specifically the response of the hypothalamic–pituitary–adrenal axis (HPA) (Möstl and Palme, 2002; Palme, 2005), since elevated concentrations of these steroids, i.e. above basal levels, may be a sign of stress. It is important to note, however, that the primary functions of these steroids are not a direct response to stress factors but rather one component of a complex set of physiological and behavioural responses to these factors (MacDougall-Shackleton *et al.*, 2019).

*Saltator similis* (Green-winged Saltator), is a passerine measuring up to 20 cm in length and weighing ~45 g, with no apparent sexual dimorphism (Sick, 2001). This species is widely distributed across South America, occurring in Argentina, Bolivia, Brazil, Paraguay and Uruguay (Sick, 2001; Birdlife, 2018). It is classified as ‘low concern’ by the International Union for Conservation of Nature (Birdlife, 2018), but this species has the highest seizure rates according to environmental organizations in Brazil (Destro *et al.*, 2012; Costa *et al.*, 2018). The Green-winged Saltator is characterized by a distinct song, contributing to its popularity among breeders in Brazil, often participating in competitions focusing on song duration (Trajano and Carneiro, 2019). In São Paulo state, these popular events occur during the birds’ breeding season, typically between September and January.

Given the importance of preserving the physical and psychological health of animals kept under human care (Alrøe *et al.*, 2001; Broom and Molento, 2004; Bayvel and Cross, 2010), this study aims to assess whether males participating in song competitions exhibit higher levels of glucocorticoid metabolites (GCM) in their excreta and whether there is a cumulative effect of steroid levels between events. We hypothesized that adult males of *S. similis* experience increased adrenal activity during song competitions. To investigate this, we measured GCM levels using an enzyme immunoassay (EIA) validated in this study for the detection of these metabolites in the studied species.

## Materials and Methods

### Ethical authorizations

The procedures outlined in this study received approval from the ethics committee regarding the Use of Animals—CEUA of FMVZ-USP (process No. 5498220318) and were authorized by SISBIO/IBAMA (No. 10013-1). Furthermore, the activities carried out at the Centro de Manejo e Conservação de Animais Silvestres (CeMaCAs) were approved and/or authorized under process No. 6027.2019-0001450-9. The identification of birds under the care of breeders was facilitated through the rings registered in the federal electronic ‘*Sistema de Gestão de Criadores Amadores de Pássaros Nativos* (SISPASS)’. This registration provided confirmation of the life histories of the animals, along with details about the number of licences issued for their participation in tournaments authorized by the *Divisão da Fauna Silvestre do Estado de São Paulo* (DEFAU). No singing competitions were organized or conducted by the authors of this study. Uro-faecal samples were collected from the birds during voluntary participation by the bird breeders, who authorized the data collection, by signing an agreement where they declare their understanding of the research.

### Animals and housing

This study utilized uro-faecal samples collected from 20 adult males of *S. similis*. Ten individuals were exclusively dedicated to immunoassay validation studies (Validation Group), while the remaining 10 were allocated to assess the impact of singing competitions (Aviary Group). Prior to conducting the tests, a veterinary assessment was conducted on the animals, confirming their health status through serological and coproparasitological laboratory tests. Furthermore, the sex of the individuals was confirmed through PCR methods utilizing blood samples. All animals had access to water *ad libitum* and were provided with a daily diet comprising a mixture of seeds, extruded feed and fruit.

The physiological validation of the EIA involved adrenal stimulation with synthetic ACTH in birds housed at CeMaCAs (23°24’43” S 46°47’29” W). These birds were placed individually in wire cages (22 × 42 × 40 cm) within a shared room. In the aviary group, animals were accommodated in separate installations, aiming to maintain consistency in sanitation practices, frequency and feeding types across all facilities. The aviary group comprised six males participating in competitions and four in the control group. Individual cages (28 × 80 × 40 cm) housed these males, with those designated for competition kept in separate rooms to ensure acoustic and/or visual isolation from conspecifics, following typical aviary management practises. All animals in the aviary group possessed SISPASS-regulated Leg Bands, and their identification numbers and dates of birth are detailed in Table 1.

**Table 1:** Competition-participating and non-participating birds

	Song duration contestant individual						Non-participating individual			
Male	T1	T2	T3	T4	T5	T6	C1	C2	C3	C4
Leg band	IBAMA OA 3,5 318 712	SISPASS 3,5 PR/A 006946	MZ 219442 3,5 06/07–004	IBAMA OA 3,5 587 738	PRA 594 3,50 809	IBAMA OA 3,5 226 870	SISPASS 3.5 SP/A 041448	IBAMA OA 3,5 395 755	SISPASS 3.5 SP/A 001460	SISPASS SP/A 3,5 056 918
Birthdate	22 August 2008	27 January 2014	5 October 2007	23 June 2010	8 November 2009	25 August 2011	06 February 2015	05 April 2011	16 April 2014	22 April 2015

### Uro-faecal material collection

A pool of uro-faecal material was collected from each individual at 4-h intervals (08:00, 12:00, 16:00, 20:00, 24:00 and 04:00). Due to logistical reasons, collection at 24:00 and 04:00 was not feasible for the animals in the aviary group. Samples from the validation group were collected continuously over 4 days at the following times: −24, −20, −16, −12, −8, −4, 0, 4, 8, 12, 16, 20, 24, 28, 32, 36 and 40 h, where 0 corresponds to the time of ACTH and saline treatments administration, as explained in the EIA validation section.

The samples of uro-faecal material from the aviary group were collected from all individuals at four different times: Thursday, designated as a rest day (Day-3); Saturday, the day before the competition (Day-1), during which the males are prepared for the event and visually exposed to females; Sunday, the day of the competition (Day 0), encompassing activities such as transporting the animals to the event site, a brief exposure to females just before the competition, and acoustic confrontations between males; and finally, Monday, the day after the event (Day 1). Considering the metabolic delay in GCM excretion occurring between 4 and 8 h after pharmacological stimulation with ACTH (as indicated by the results in the physiological validation section for the species under study and the provided feeding conditions), the daily-collected samples are reflective of the evaluated day’s events. Therefore, transportation activities, typically occurring at 05:00, and competition activities, commencing at 08:00, are anticipated to be manifested in the subsequent 12:00 collections.

### Extraction of glucocorticoid metabolites

The extraction of GCM followed the method outlined by Barbosa-Moyano *et al.* (2019) with some modifications. Briefly, after lyophilizing the samples using an L108 lyophilizer, 0.2 g of material were weighed and then diluted in 5 ml of 80% methanol. Each mixture underwent three 5-min shaking cycles in a multivortex at 3500 rpm and was subsequently centrifuged for 15 min at 1500 g. Following centrifugation, 500 μl of the supernatant were transferred to a new glass tube, and the solvent was evaporated under a water bath (60°C) and air flow. After drying, 250 μl of 100% methanol were added, vortexed and the samples were stored at −20°C until hormone assays were conducted. Before performing EIAs, methanol was removed by air drying, and 0.3 ml of a buffer solution (NaH_2_PO_4_, Na_2_HPO_4_, NaCl, BSA) was added to the tubes.

### Analytical and physiological validation of the enzyme immunoassay

The quantification of GCM levels was conducted using corticosterone EIA CJM006 from the UC Davis Endocrinology Laboratory, employing corticosterone conjugate (HRP) as a marker. The specificity of the assay was initially assessed through the parallelism test, where a pool of extracts (diluted from 1:2 to 1:128 in EIA BSA buffer: Na_2_HPO_4_ 0.1 M; NaH_2_PO_4_ 0.1 M; NaCl 0.15 M cm 0.1% BSA, pH 7.0) was compared to a serial dilution of the corticosterone standard (Corticosterone Crystalline C2505 Sigma-Aldrich). Accuracy and matrix interference were evaluated by spiking previously quantified samples with known amounts of the corticosterone standard. The precision of the EIA was determined by calculating intra-assay and inter-assay coefficients of variation (CVs). The mean intra-assay CV was computed for duplicates of samples within each plate, and the inter-assay CV was calculated for high (30%)- and low (70%)-binding controls. ELISA plates were read with an ELISA spectrophotometer (ELx 808TM Bio Tek Instruments Inc, USA) at an absorbance of 405 nm.

The physiological validation of the EIA included adrenal stimulation with synthetic ACTH in adult males of the studied species. To achieve this, the birds in the validation group were randomly assigned to three experimental subgroups: Group A (*n* = 3) received a dose of 0.5 mg/kg of ACTH (Synacthen®, Sigma Chemical No. A-6303), Group B (*n* = 3) received a dose of 0.25 mg/kg of ACTH, and Group C (*n* = 4) received 0.2 ml of saline solution (0.9% NaCl). The intervals and duration for uro-faecal material collection are described above.

### Singing competition

Each singing competition in the duration modality involves up to 100 birds of the species *S. similis*, individually housed in cages (22 × 42 × 40 cm) placed ~0.20 m apart from each other. These cages are supported on 1.5-m-high tripods, forming a circle that facilitates visual and acoustic exposure among the participating males. The competitions typically occur in sports centres or arenas between 08:00 and 12:00 h at room temperature. Each bird undergoes two rounds of evaluation by a judge, with each assessment lasting 15 and 10 min, occurring ~10:00 and 11:30. The winning bird is determined by the highest score, based on the most songs performed during these two time slots. The total exposure time between birds can extend up to 4 h per event, depending on the total number of participants in the tournament. The animals under evaluation were observed over three competitions, spaced between 1 and 4 weeks apart, contingent upon the availability of these events. Preceding the tournaments, males are visually exposed to a female for ~30 min, chosen based on each male’s preference. It’s essential to note that, as reported by the bird breeders, males brought to the competition are generally not mated, as this may impact their performance. These females are typically also brought to the tournaments and kept in a separate room from the males during the event.

### Data analysis

For the analytical validation of the EIA, specificity was indirectly assessed by testing the parallelism between the linear and angular coefficients of the curves resulting from the dilution of the corticosterone standard versus the dilution of the pool of Green-winged Saltator samples. A *t*-test was performed after adjusting the data in Log10. The percentage accuracy of the EIA was determined by dividing the observed concentration by the expected concentration and multiplying this value by 100. In the physiological validation, the basal average of GCM production for each animal was calculated from the values reported in the samples collected prior to the administration of the treatments (ACTH and saline solution). The peak value of these steroids was determined when the sample value exceeded the individual basal average plus two times the standard deviation value. Additionally, a generalized linear model (GLM) was applied, followed by Tukey’s *post hoc* test, to compare the levels of GCM between Groups A (doses of 0.5 mg/kg ACTH), B (dose of 0.25 mg/kg ACTH) and C (saline solution) of the samples taken between 0 and 20 h.

GCM concentrations in male participants and non-participants in tournaments were presented as mean values in nanogrammes per gramme units, along with their standard deviation. After transforming the data according to the trend, GLM was applied, followed by Tukey’s *post hoc* test. Initially, the levels of GCM were compared between male participants and non-participants in the aviary group, considering the sampling day as an independent variable. Subsequently, GCM levels of competition-participating males were evaluated according to sampling day (D-3, D-1, D0 and D1) and the number of tournaments (1st, 2nd and 3rd), as well as interactions between these factors, using GLM followed by Tukey’s *post hoc* test. All data were analysed using Statistical Analysis System software (version 9.3—SAS, Windows, USA), with a significance level of 95% (α ≤ 0.05).

## Results

### Analytical and physiological validation of the enzyme immunoassay

**Figure 1:**
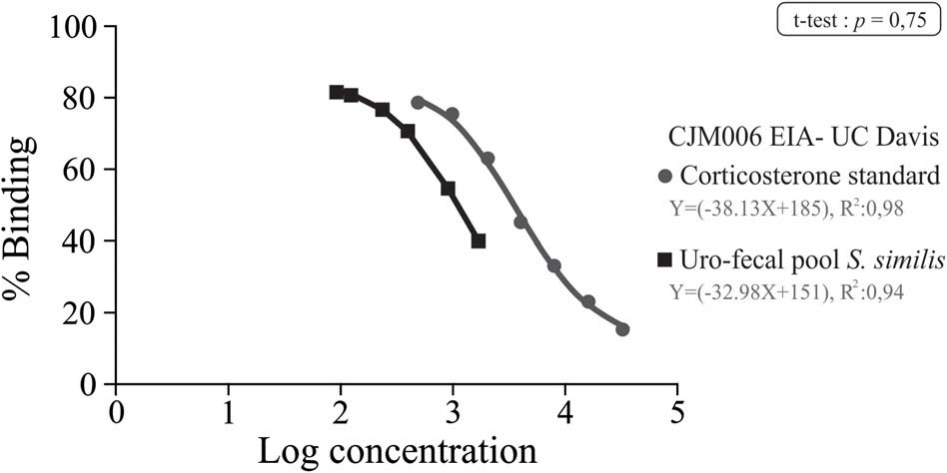
Parallelism of GCM levels in uro-faecal material detected in Green-winged saltator using direct corticosterone EIA. The standard curve (circle) follows the equation Y = −38.13X + 185, with an r^2^ value of 0.98. The uro-faecal material extract (square) exhibits a linear relationship described by Y = −32.98X + 151, with an r^2^ value of 0.94.

The angular coefficient of the *S. similis* pool curve (slope = −38.13 ± 2.33) was not significantly different from the corticosterone standard curve (slope = −32.98 ± 4.03, *P* = 0.75), confirming the immunogenic resemblance between the antigens in the standard and those present in the bird samples (Fig. 1). The EIA demonstrated an accuracy of 96.07% (± 3.16), with intra-assay and inter-assay coefficients of variation of 5.08 and 8.24%, respectively.

**Figure 2:**
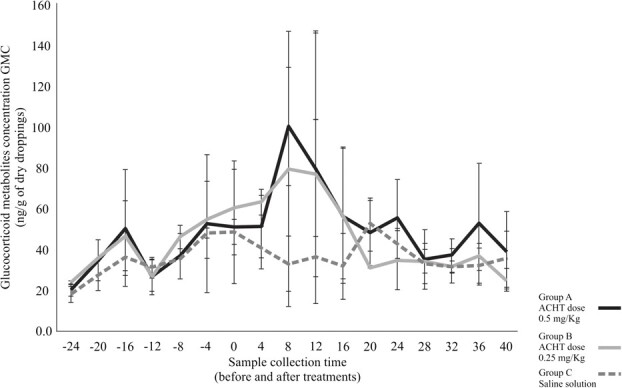
Concentrations of GCMs extracted from freeze-dried uro-faecal samples of *S. similis* males over four consecutive days of collection, with a collection interval of 4 h (time from −24 to 40 h). The reference point ‘0’ marks the initiation of treatment administration (saline solution and/or ACTH analogue). The black and grey solid lines depict GCM concentrations in males treated with ACTH analogue at doses of 0.5 and 0.25 mg/kg, respectively, while dotted lines represent GCM concentrations in animals treated with saline.

A total of 157 uro-faecal samples underwent processing and evaluation in the physiological validation of the EIA. Figure 2 illustrates the average concentrations of GCMs in Green-winged Saltators, along with their respective standard deviations, obtained from samples collected 24 h before the treatments with ACTH and saline (Time 0) and up to 40 h after the injection. Uro-faecal samples collected between the ACTH and saline treatments at Time 0 and up 20 h post-injection exhibited average GCM values of 65.75 ± 42.38 ng/g dry weight for Group A, 65.43 ± 48.03 ng/g for Group B and 39.81 ± 14.41 ng/g for Group C (saline), confirming a significant difference in GCM levels between Group C and Groups A and B [F = 3.37, *P* = 0.0421]. Peak GCM values were exclusively observed in ACTH-treated animals, recorded in the uro-faecal material pool collected 4 and 8 h after adrenal stimulation. Among these animals, samples collected 12 h after the pharmacological challenge did not exceed twice the standard deviation, except for one male in Group B, which maintained values above the baseline average until the collection 16 h after stimulation.

### Variation in GCM between contest and non-participant groups

**Figure 3:**
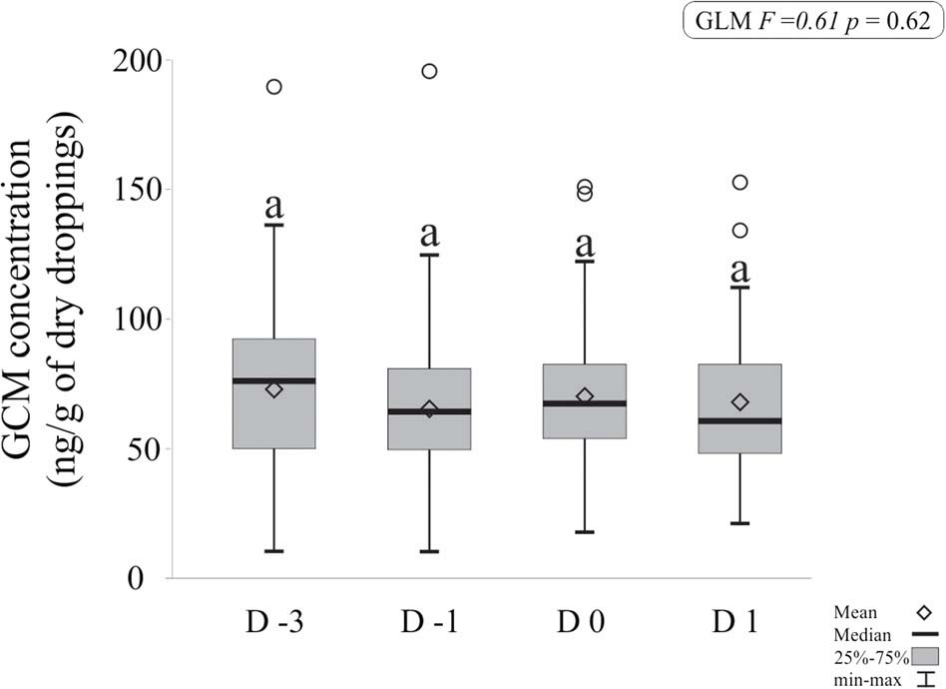
Levels of GCMs in uro-faecal material from males not participating in song competitions across four distinct periods—Rest (D-3), Pre-competition (D-1), Competition (D0) and Post-competition (D1). The boxes emphasize the data distribution, including median and quartiles, while the whiskers delineate the range of values. Values sharing the same letter indicate no statistically significant differences (Tukey *post hoc*).

A total of 459 uro-faecal material samples were collected, comprising 271 from males participating in the singing competition and 188 from males in the control group. Males engaged in the tournament exhibited lower mean GCM values (33.43 ± 22.09 ng/g) compared to their non-participating counterparts (70.09 ± 29.45 ng/g) [F = 2.46, *P* = 0.01], and this difference was confirmed by Tukey’s test. The GCM values of non-participating males showed no significant differences between collection days [F = 0.61, *P* = 0.62], between evaluated weeks [F = 0.49, *P* = 0.63] or in the interaction of these factors, as illustrated in Fig. 3.

**Figure 4:**
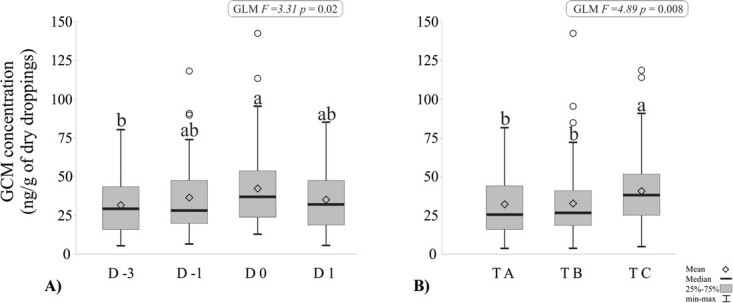
Levels of GCMs in the uro-faecal material of males participating in tournaments. (A) Distribution of GCM levels across four different periods—Rest (D-3), Pre-competition (D-1), Competition (D0) and Post-competition (D1). (B) Distribution of GCM levels across three singing tournaments. Values with the same letter indicate no statistically significant differences (Tukey’s *post hoc*).

Significant differences in GCM levels were observed between collection days [F = 3.31, *P* = 0.02] and among evaluated events [F = 4.89, *P* = 0.00 (*P* < 0.01)] in males participating in the song competition, but not in the interaction between these factors [F = 1.43, *P* = 0.20]. Tukey’s test, conducted between collection days, confirmed that GCM levels on the competition day (D0 = 38.29 ± 26.12 ng/g) were 1.34 times higher compared to the rest day (D-3 = 28.64 ± 17.86 ng/g), as illustrated in Fig. 4a, indicating a significant difference between these 2 days. In contrast, no significant differences were observed between pre-competition (D-1) and post-competition (D1) days. Additionally, GCM levels were significantly higher in the third competition event (TC = 38.84 ± 23.06 ng/g) compared to the values recorded in Tournaments A (30.25 ± 19.18 ng/g) and B (30.85 ± 22.92 ng/g).

## Discussion

During the song competition, the Green-winged Saltator exhibited an increase in their levels of GCMs, indicating heightened activity in the HPA axis. This aligns with the acute stress response observed when confronting congeneric males. In the analytical validation of EIA CJM006 for quantifying GCM in Green-winged Saltator males, the parallelism test revealed similar behaviour in both the dilutions of the uro-faecal material pool and the dilutions of the corticosterone standard used. This confirms the suitability of the immunoassay for assessing concentrations of the studied hormone, in this case, GCMs (Brown *et al.*, 2005; Touma and Palme, 2005; Barbosa-Moyano *et al.*, 2024b). The precision rate and accuracy percentage of the quality parameters are notable, with intra- and inter-assay coefficients consistently <10%, aligning with literature findings (Brown *et al.*, 2005; Lèche *et al.*, 2009; Watson *et al.*, 2013; Barbosa-Moyano *et al.*, 2019). Results from the titration test for corticosterone using the EIA technique produced a curve adhering to expected standards, with r^2^ values close to one (Brown *et al.*, 2005; Touma and Palme, 2005; Sinhorini *et al.*, 2020). These quality parameters are crucial for ensuring the suitability of the dosage method in identifying the expected metabolite, with no observed underestimation or overestimation of results (Brown *et al.*, 2005). The CJM006 immunoassay’s efficacy for non-invasive monitoring of adrenal activity has been previously validated across a diverse range of taxa, including birds, reptiles, amphibians and mammals (Watson *et al.*, 2013).

The administration of synthetic ACTH to stimulate glucocorticoid production proved effective in establishing a cause-and-effect relationship between the induction and excretion of GCMs, thereby physiologically validating the EIA. Inter-individual differences in baseline GCM concentrations observed in this experiment for peak calculation were consistent with findings in other studies (Ferreira *et al.*, 2015; Barbosa-Moyano *et al.*, 2003). The peak GCM values observed in individuals from the groups injected with ACTH during physiological validation confirm that monitoring adrenal activity in the Green-winged Saltator can be achieved by measuring these metabolites. Additionally, the two doses of ACTH administered to the groups both resulted in peak GCM values in the excreta of the birds. This response to hormonal stimulation, followed by the normalization of GCM levels, has also been demonstrated in other studies with passerines, such as the European Stonechat *Saxicola torquata rubicola* (Goymann *et al.*, 2002a) and the Chestnut-bellied Seed-Finch *Sporophila angolensis* (Barbosa-Moyano *et al.*, 2019). The time interval between the administration of synthetic ACTH and the increase in GCMs in faeces varies among species and can even be influenced by the type of feeding. For instance, in passerines, the intraperitoneal application of synthetic ACTH in male European Stonechat results in a significant increase in GCM between 3 and 6 h after administration (Goymann *et al.*, 2002b), similar to the findings in the Green-winged Saltator. However, in other species, such as the Chestnut-bellied Seed-Finch, it has been observed that the peak of GCM excretion occurs 12 h after injection (Barbosa-Moyano *et al.*, 2019). Given that most of the males studied showed a delay of between 4 and 8 h, we conclude that the samples collected up to 20:00 reflect the events of the day evaluated, especially those corresponding to the day of the tournament. The administration of saline solution did not increase GCM levels in Green-winged Saltator, following patterns observed in other studies with birds, such as the Domestic Fowl (*Gallus gallus*)*,* the Great Cormorant (*Phalacrocorax carbo*), Eurasian Goshawk (*Accipiter gentilis*) (Dehnhard *et al.*, 2003), the Mourning Dove (*Zenaida macroura*) (Washburn *et al.*, 2003) and Budgerigar (*Melopsittacus undulatus*) (Young and Hallford, 2013).

Males not participating in song competitions exhibited higher levels of GCM compared to contest males; however, those belonging to this group did not show variation between sampling days and weeks. One possible explanation for these animals showing elevated GCM levels may be inherent temperament and/or genetic characteristics (Cockrem, 2013, 2022; Barbosa-Moyano *et al.*, 2019; Barbosa-Moyano and Oliveira, 2003). Another relevant aspect to consider is the management implemented in aviary facilities, as these animals are constantly exposed visually and audibly to congeners of the same and/or different sex. This situation warrants further research, particularly to assess the impact on animal welfare when individuals are kept near animals widely considered to be territorial. Studies on birds by Carere *et al.* (2003) and Landys *et al.* (2010) demonstrate that territorial challenges can elevate corticosterone levels, suggesting that prolonged exposure to territorial individuals may increase stress and negatively affect welfare. Although *S. similis* is considered territorial, we are not aware of any published research supporting this behaviour. However, this knowledge is widely recognized by breeders, veterinarians and biologists in rescue centres. For example, specialists at the CeMaCAS rescue centre observed that wild males are susceptible to capture when a captive male is introduced into the territory (placed inside a trap-cage), leading to a confrontation between birds and the capture of the free-living individual.

In the context of the singing competition, the discerned elevation in GCMs among the evaluated Green-winged Saltator implies an acute stress response (McEwen and Sapolsky, 1995; Wingfield and Sapolsky, 2003). Similar observations were made in other species such as the Great Tit (*Parus major*) (Carere *et al.*, 2003), Greylag Goose (*Anser anser*) and Domestic Geese (*Anser domesticus*) (Hirschenhauser *et al.*, 2005), where an increase in glucocorticoid levels is foreseeable during social interactions or the establishment of reproductive territories (Romero *et al.*, 1998). Song competitions are systematically arranged during the species’ breeding season, and the consequent adrenal response is considered a normal physiological reaction. The territorial song, emblematic of aggressiveness and/or dominance, becomes particularly pronounced when a male engages with another of his species, a behaviour observed consistently throughout competitions (Gil and Gahr, 2002; Lyra *et al.*, 2022). The opponents’ singing during competitions serves as challenging stimuli that can induce allostatic overload in individuals (Landys *et al.*, 2006; McEwen and Wingfield, 2010). Despite the observed increase in GCM levels on the days of the competitions (D0), it is evident that the evaluated animals reduced GCM levels in the samples collected on the following day (D1), reaching levels that were not statistically different from the rest days (D-3). This observation preliminarily suggests that the well-being of the animals may not have been compromised. Nevertheless, it is imperative to underscore that a holistic evaluation of animal welfare necessitates the integration of comprehensive behavioural assessments of affective states (Beaulieu, 2024), along with other tests of biological functioning, including indicators of immune function (Davis *et al.*, 2008; Davis and Maney, 2018).

Logistical constraints limited our evaluation to three song competition events. However, a significant observation emerged: an increase in the number of tournaments corresponded to elevated levels of GCMs. This finding prompts crucial considerations for future research, suggesting that custodians of these animals may benefit from participating in fewer events per year. It is essential to bear in mind that prolonged exposure to stress can precipitate physiological problems, such as weight loss, alterations in the immune system and diminished reproductive capacity (Fischer and Romero, 2019). The authors of this article strongly advocate for further research that seamlessly integrates the assessment of both immune and behavioural activity, comparing wild birds with those raised under human care, as demonstrated in other bird species (Vidal *et al.*, 2019). In this context, key considerations encompass questioning the frequency of tournaments per year, evaluating the optimal length of these events, determining the appropriate number of individuals participating, addressing transportation regulations and exploring the implications of keeping males in the same breeding environments. To ensure the welfare of the animals involved, there is a pressing need for effective regulation and monitoring during tournaments by the respective government bodies of each country. This regulatory framework is crucial not only for overseeing the well-being of the animals but also for preventing any unlawful activities that may be attracted by these events.

## Conclusion

This study successfully validated an EIA for quantifying GCMs in the Green-winged Saltator, both physiologically and analytically. Additionally, it confirmed the feasibility of non-invasively monitoring the adrenal response in males of this species. The evaluation of singing competitions on participating males revealed an increase in GCM levels, indicating an elicited acute stress response. It is crucial to note that this elevation may not necessarily imply a compromise in the animals’ well-being. Therefore, it is imperative to incorporate other behavioural and laboratory assessments to provide a comprehensive understanding of this issue.

## Data Availability

The data that support the findings of this study are available on request from the corresponding author.
